# Temperature effects on egg and larval development rate in European smelt, *Osmerus eperlanus*, experiments and a 50 year hindcast

**DOI:** 10.1111/jfb.14314

**Published:** 2020-04-05

**Authors:** Marieke Keller, Pieke Molenaar, Joep de Leeuw, Wolf Mooij, Adriaan Rijnsdorp, Karen van de Wolfshaar

**Affiliations:** ^1^ Aquaculture and Fisheries Group Wageningen University Wageningen The Netherlands; ^2^ WMR, Wageningen Marine Research, Wageningen UR IJmuiden The Netherlands; ^3^ Department of Aquatic Ecology Netherlands Institute of Ecology (NIOO‐KNAW) Wageningen The Netherlands; ^4^ Department of Aquatic Ecology and Water Quality Management Wageningen University Wageningen The Netherlands

**Keywords:** Arrhenius temperature, degree day, IJsselmeer, incubation experiment, multinomial model

## Abstract

This study investigates the effect of water temperature on the development rate of eggs and larvae, the duration of the endogenous feeding period and its consequences for recruitment of smelt (*Osmerus eperlanus* *)* in Dutch lakes IJsselmeer and Markermeer. This study measured temperature‐dependent egg and larval development rates as well as mortality rates from fertilization till the moment of absorption of the yolk‐sac and from yolk‐sac depletion onwards in temperature‐controlled indoor experiments. Using multinomial modelling the authors found significant differences in development time of egg development stages under different temperature regimes. Based on historic water temperatures, the model predicted that the larval endogenous feeding period has advanced at a rate of about 2.9 days per decade in a more than 50 year period since 1961, yet there was no change in the duration of the endogenous feeding period. As zooplankton is more responsive to daylight than water temperature cues, a mismatch between the peak of the onset of exogenous feeding of smelt and the peak of zooplankton blooms could lead to high mortality and therefore low recruitment of smelt. Such a mismatch might contribute to a decline in the smelt population in Lake IJsselmeer and Lake Markermeer.

## 1 INTRODUCTION

European smelt, *Osmerus eperlanus* (henceforth referred to as smelt), is a small salmonid species that inhabits coastal and landlocked waters in Western Europe (Wheeler, 1969). The biomass of the smelt population in Lake IJsselmeer and Lake Markermeer is of importance for fisheries and nature conservation (Mous *et al*., 2003). Smelt is exploited in a directed fishery, which is managed by a licence system in combination with a 3‐week dispensation during the otherwise‐closed season. This dispensation is based on a threshold value of the smelt biomass index determined from autumn surveys (de Leeuw *et al*., 2008). In addition, smelt is a key species in the ecosystem of both lakes, being a consumer of zooplankton and important food source for fish‐eating birds as well as the commercially exploited eel (*Anguilla anguilla*), perch (*Perca fluviatilis*) and pikeperch (*Sander lucioperca*) (Buijse *et al*., 1993; Mous *et al*., 2003). The number of fish‐eating birds dependent on smelt has declined, and the breeding success of common tern (*Sterna hirundo*) is strongly reduced in years with low abundance of smelt (van der Winden *et al*., 2019). Although the smelt population is of ecological and economic importance, its biomass has decreased over the years, leading to a closure of the fisheries in recent years.

Since the late 1980s smelt population has declined considerably (de Leeuw *et al*., 2008). This decline in smelt coincided with changes in several external factors: an increase in landings by the spring smelt fishery (Mous, 2000), a management‐driven reduction in nutrients (de Leeuw *et al*., 2008) and a climate‐driven increase in average water temperature (Mooij *et al*., 2008). The latter observation raises the question to what extent this increase may have been contributing to the decline in smelt populations (Noordhuis, 2010). In Lake Peipsi and in the River Thames estuary high water temperatures and reduced oxygen concentrations appeared to have been detrimental to smelt populations (Kangur & Kangur, 2007; Power & Attrill, 2007). In more northern regions smelt recruitment is suggested to depend on food availability and predator presence and less on temperature, although summer temperature plays an important role in growth and survival of older smelt (Nyberg *et al*., 2001). Power and Attrill (2007) suggest that temperature is important during early development of smelt, and O'Brien *et al*. (2012) report earlier spawning with high spring temperatures for rainbow smelt (*Osmerus mordax*).

Although little is published on smelt larval development related to temperature in particular, information from other species is available. Changes in water temperature are known to affect the phenology and survival of early life history stages of fish and therefore recruitment (Edwards & Richardson, 2004; Rijnsdorp *et al*., 2009; Thackeray *et al*., 2010; Winder & Schindler, 2004). An increase in water temperature may affect the recruitment process of smelt in multiple ways: it may affect the timing of spawning and may also influence the developmental rate of the eggs and larvae, thereby influencing the duration of the endogenous feeding period and the onset of exogenous feeding. Inspired by Hjort (1914), who emphasized the importance of the first exogenous feeding phase for the survival of fish larvae and the subsequent recruitment (critical period hypothesis), Cushing (1990) discussed the importance of the match between the timing of the occurrence of the fish larvae and their planktonic food for larval survival (match–mismatch hypothesis). Platt *et al*. (2003) showed that the survival of haddock larvae was significantly related to temperature‐driven variation in the timing of the spring bloom in phytoplankton.

Because there is little known about the larval development rates of smelt, the relationship between larval development of smelt and temperature was studied. The objectives of this study were (a) to experimentally assess the developmental rates of smelt larvae from egg to first feeding with laboratory‐spawned batches of smelt eggs and quantify temperature dependence of the development rate of eggs and the endogenous feeding period of yolk‐sac stage of larvae and (b) to assess the effect of historical changes in temperature on development under natural conditions in the Lake IJsselmeer area. The results of the experiments were used to parameterize a statistical model which was used for the second objective of the study to assess the rates from an historical perspective using lake temperature data. It was hypothesized that the development rates have increased with warmer temperatures in the past 50 years. Implications for smelt population dynamics and possible mechanisms for temperature‐dependent declines observed in smelt stocks were discussed.

## 2 MATERIALS AND METHODS

### 2.1 Study area

**FIGURE 1:**
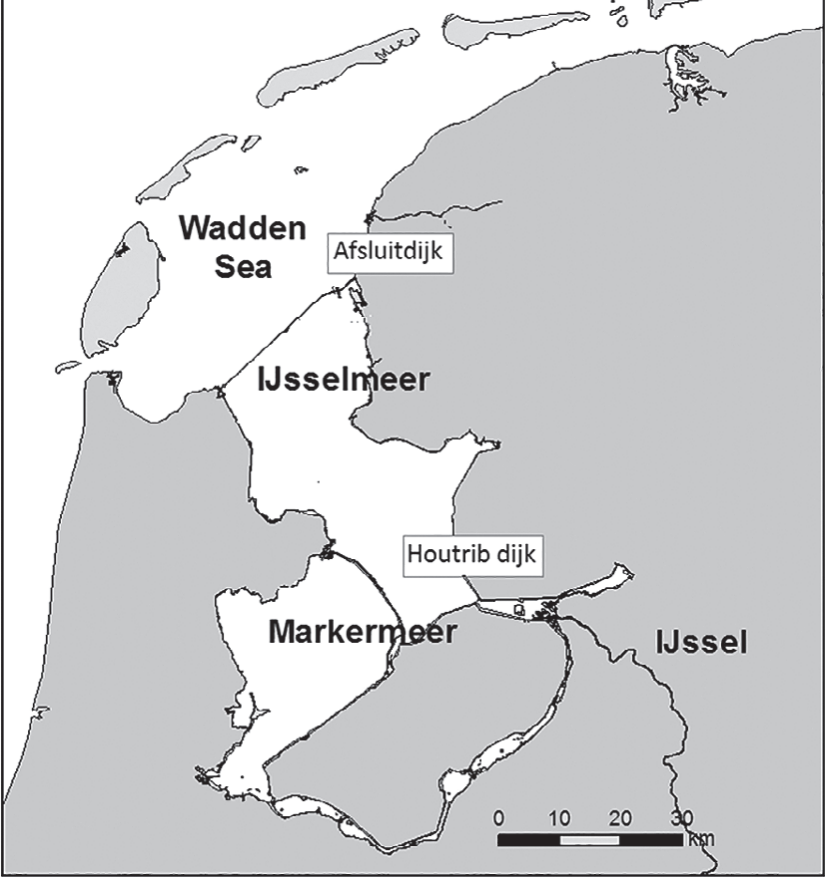
Map of the study area showing the dykes separating the lakes from the Wadden Sea and each other

In 1932 the estuary “Zuiderzee” was separated from the Wadden Sea by the construction of 32 km dyke, called the “Afsluitdijk,” creating Lake IJsselmeer (Figure 1). In 1975, a second dyke, the “Houtribdijk” was built resulting in the creation of Lake Markermeer with a surface area of 700 km^2^ and a mean depth of 3.2 m. The current Lake IJsselmeer has a surface area of 1225 km^2^ and a mean depth of 4.5 m and discharges water from the River IJssel to the Wadden Sea.

Both lakes are polymictic throughout the year, although short‐term stratification may occur during warm days with little wind. From 1961 to 2006, water temperatures in both lakes increased over time by 0.015°C year^−1^, resulting in a total increase of almost 0.7°C (Mooij *et al*., 2008).

There is little known about movements of smelt between Lake IJsselmeer and Lake Markermeer through the sluices in the “Houtribdijk,” but the exchange between the lakes is considered low. Wash‐outs of smelt at discharges from IJsselmeer into the Wadden Sea are common (Noordhuis, 2010), but there is little or no migration of smelt from the Wadden Sea to Lake IJsselmeer (Tulp *et al*., 2013).

### 2.2 Spawning population in the laboratory

Fish with an average length of 71 mm were collected just before the spawning season of 2011, on 28 February, in Lake IJsselmeer in two 5 min tows with a 4 m small‐meshed pelagic beam trawl (5 mm cod end mesh) at a speed of 1 knot with a small research vessel *Tsjûkemar*. The catch was directly transferred into a barrel containing 25 l of lake water mixed with a light anaesthetic (1.5 ml ethylene glycol monophenyl with 138.16M). Within 3 h, the fish were transported to the laboratory, where they were divided in two groups of 80 individuals each and placed in two 150 l polyester tanks filled with fresh water and a 20% replacement per day. Each group was acclimatized for 21 days in a climate chamber at 6.0°C. This temperature is comparable to the water temperature in Lake IJsselmeer during this time of year. Light regime was adjusted to mimic daylight hours (started at 11 h and increased with 15 min twice a week). Light intensity at the water surface during acclimatization was set at 5 lx. Each group of fish was fed twice daily with approximately 35 g of a mix of defrosted white (*Chaoborus spp*.) and red mosquito larvae (*Chironomus plumosus*), live brine shrimp (*Artemia spp*.), live white mosquito larvae and live *Daphnia spp*.

Directly after capture and transport 39 fish died (24%; 39/160). Thereafter 9 fish (7%; 9/121) died during the 21‐day acclimatization period, 6 died during the spawning events (5%; 6/112) and another 9 fish (8%; 9/106) died in the 2 weeks after spawning but not related to spawning.

Spawning was induced on 30 and 31 March and on 4 and 6 April 2011, by increasing the water temperature by 3° over a period of 24 h, increasing light intensity to 1005 lx (light of an overcast day) and a regularly interrupted water current. Spawning occurred in the night after the change in temperature, light and current conditions. Before spawning the bottom of the spawning tanks was equipped with 10 glass plates for collecting the adhesive eggs. A nylon twisted rope with an unravelled end was placed on the glass plates as an artificial spawning substrate. Eggs did stick to the added glass plates and metal heaters in spawning tanks, but not to the polyester tank walls and substrate ropes. After spawning the adult fish were returned to the lake where they were caught. This research was performed in compliance with Dutch law in force on animal ethics. No surgical procedures were performed.

### 2.3 Experimental set‐up

An incubation experiment was performed in six 65 l aquaria, which were together placed in a climate chamber with air temperature set at 6°C. Three temperature treatments were applied (6, 9 and 12°C), which were replicated (Table 1). The two replicates of the 6°C treatment were cooled by the air in the climate chamber, whereas the replicates of the 9 and 12°C treatments were heated with 300 W heaters connected to an adjustable thermostat to establish stable temperatures (±0.16 °C averaged over all aquaria, Table 1). The heated aquaria were insulated with polystyrene insulation material to prevent heat exchange among the aquaria. A flow‐through system provided fresh water at a rate of 1 lh^−1^. The water was kept aerated to a saturated oxygen level. Fluorescent lighting was placed above the aquaria providing a light intensity of 912 lx at the water surface.

**TABLE 1:** Total number of eggs placed in each aquarium to determine the developmental rate (larval mortality window), replicated at three temperatures

		Aquarium 1	Aquarium 2	Aquarium 3	Aquarium 4	Aquarium 5	Aquarium 6
Average °C		12.1	12.1	9.1	9.0	6.1	5.7
s.d. (±)		0.1	0.1	0.1	0.2	0.2	0.3
Batch 1	*N*	773	128	392	277	324	198
Batch 2	*N*	118	113	258	519	107	87

*Note*: Batch 1 denotes the spawning event in March and Batch 2 that of April.

### 2.4 Egg and larval sampling

The glass plates with fertilized eggs from the spawning tanks were horizontally placed at the bottom of six aquaria prepared for each temperature treatment (Table 1). The eggs of each spawning event (either March or April), further denoted as “batch,” were allocated randomly to an experimental aquarium for further development.

**FIGURE 2:**
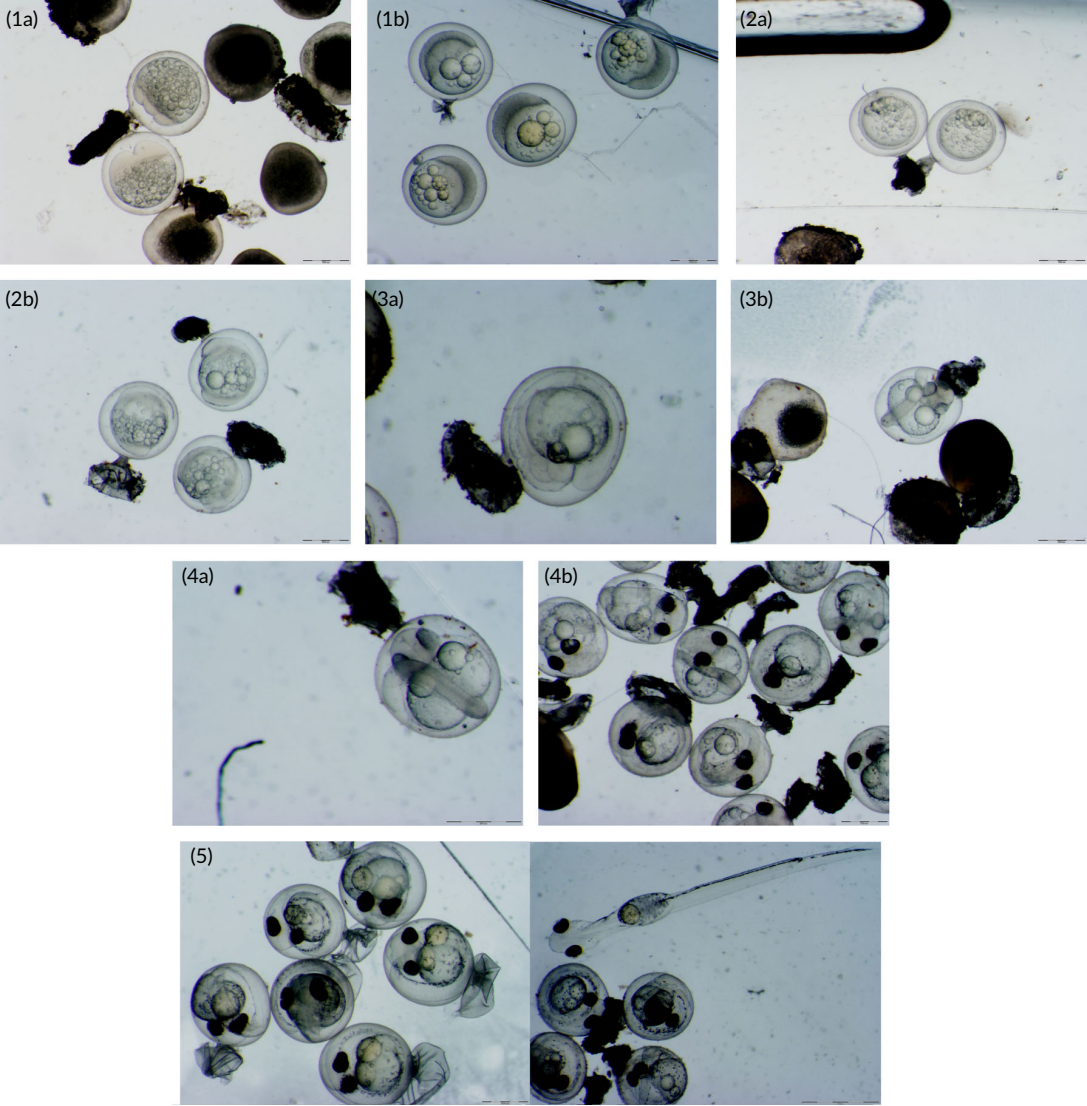
Redefined egg development stages used as a reference to classify sampled eggs. Stage 1a: direct after fertilization; stage 1b: gastrulation up until the epibolia; stage 2a: start somitogenesis; stage2b: optic cups, Kupfer's vesicle development; stage 3a: embryo surrounding the yolk completely; stage 3b: first pigmentation of the eyecups; stage 4a: closed pigmentation of the optic cups; stage 4b: cavity for intestinal canal, first melanophores on yolk sac; stage 5: melanophores along the intestines, mouth formed; Hatch: Larvae free from egg envelops. Photos taken by P. Molenaar

Egg samples to establish development were taken between 9 and 10 a.m. The first sample was taken immediately after the allocation of the batches over the temperature treatments to determine the starting point of development. At each sampling event, a minimum of five eggs (mean = 5.95 number; range = 1–10) were sampled from each batch using a flexible plastic pipette. Within 5 min eggs were put in Petri dishes containing aquarium water. Photographs were taken using a binocular microscope (Olympus SZX‐ILLK 200) equipped with a camera (Olympus DP70). Images were processed using Olympus software (Cell^Dlife science documentation software). Each sampled egg was classified individually according to the description of the developmental stages (Supporting Information; Figure 2).

Hatching rates were monitored by counting the empty egg shells twice a day until all eggs were hatched. For each batch larval mortality was recorded daily by counting and removing dead larvae.

### 2.5 Egg and larval stages

A total of 80 stages have been described for eggs of *Osmerus eperlanus eperlanus* (Gorodilov & Melnikova, 2006). For this study, the stages of egg development were combined into 10 (sub‐stages) (Molenaar, 2011) (see Supporting Information for a detailed description). In addition, a stage referring to the larval yolk‐sac stage of the now‐free‐swimming larvae was added. Next to these developmental stages two indices were assessed. The index of 50% hatching refers to the probability of half of the eggs reaching hatching and becoming free‐swimming larvae. The index of 50% mortality refers to the probability that half of the free‐swimming larvae have died.

### 2.6 Probability distributions of stage at age

Because eggs of the same age can be in different stages, a multinomial logit link model (Equation 1) was used to estimate how temperature affected development (Bernal *et al*., 2008; Geffen & Nash, 2012; Ibaibarriaga *et al*., 2007). Following Ibaibarriaga *et al*. (2007) and Bernal *et al*. (2008), the conditional probability of an egg (*P*(*stage* = *i*| *stage* ≥ *i*)) being in a certain stage *i* given that the egg is in stage *i* or higher is dependent on the rearing temperature (*T*) and age (number of days after fertilization) at the time of observation:

(1) $$\text{logit}\left(P\left[\text{stage}=i|\text{stage}\geq i\right]\right)=ɳ\left(\mathrm{age},\text{temp},\text{batch}\right)$$

where *ɳ* is a linear predictor which allows for the effects of age, temperature and batch with stage. Model selection was based on goodness of fit using the Akaike information criterion (AIC) (Table 2). The study was started with a full model containing the explanatory variables Age, Temperature and Batch and their interactions and successively eliminated non‐significant terms, starting with the interaction terms. The model with the lowest AIC was selected as the preferred model. The probability distribution of each stage was predicted with the selected model and used to calculate a number of metrics of development: (a) mean age at each stage and (b) transition points when 50% of the eggs had hatched (p50‐hatch) or died post hatching (*e.g*., yolk‐sac stage 7) (p50‐mortality). To estimate the development rate of smelt in general, the model without batch was used, again by selecting the model with the lowest AIC of all models without batch as a factor. The endogenous feeding period duration was defined as the number of days between 50% hatching and 50% mortality.

**TABLE 2:** Overview of equations for model selection and outcome of Akaike information criterion (AIC) (Equation 1)

	*df*	AIC
Model 1 (*P*_*i*_ = *α* + *β*_Age_ + *β*_Temperature_ + *β*_Batch_ + *β*_Age *Temperature_ + *β*_Age*Batch_ + *β*_Temperature*Batch_ + ε)	70	11,505.48
Model 2 (*P*_*i*_ = *α* + *β*_Age_ + *β*_Temperature_ + *β*_Batch_ + *β*_Age *Temperature_ + *β*_Age*Batch_ + ε)	60	11,564.30
Model 3 (*P*_*i*_ = *α* + *β*_Age_ + *β*_Temperature_ + *β*_Batch_ + *β*_Age *Temperature_ + ε)	50	11,687.47
Model 4 (*P*_*i*_ = *α* + *β*_Age_ + *β*_Temperature_ + *β*_Age *Temperature_ + ε)	40	11,833.33
Model 4 (*P*_*i*_ = *α* + *β*_Age_ + *β*_Temperature_ + *β*_Batch_ + ε)	40	14,244.34
Model 5 (*P*_*i*_ = *α* + *β*_Age_ + *β*_Temperature_ + ε)	30	18,725.24
Model 6 (*P*_*i*_ = *α* + *β*_Age_ + ε)	20	38,667.24
Model 5 (*P*_*i*_ = *α* + *β*_Temperature_ + ε)	20	52,284.50

*Note*: Model 1 includes the term “batch” which accounted for possible variability between eggs originating from different spawning events. *df* = degrees of freedom.

### 2.7 Effect of temperature on development

The Arrhenius temperature, which determines the temperature effect on the rate of metabolic processes, is a central parameter of bio‐energetic models such as the Dynamic Energy Budget models that are widely used to explore the effects of climate change on fish (Freitas *et al*., 2007; Pecquerie *et al*., 2009; Teal *et al*., 2012). The Arrhenius temperature (*T*_A_) was estimated by a regression of the log of the measured development rate against the inverse of the temperature (Kooijman, 2010):

(2) ln1Ageiτ=δ+TA1τ+273

where *Age*_*ij*_ is the age (days) of the developmental stage *i* at temperature *τ* obtained from Equation 1. Parameter *δ* is the stage‐specific intercept.

Then, given the estimate of *T*_A_ and the intercept as calculated earlier based on the stage‐specific development rate and ambient temperature from the experiments *τ*, the same equation can be used to calculate the developmental rate of each stage as a function of temperature (*Age*_*iτ*_).

For each development stage, the degree day (“DD,” the sum of the daily temperatures above the species‐specific temperature threshold up to that stage) required to reach that particular developmental stage was determined. This method thus converts calendar time into a measure of physiological time (Neuheimer & Taggart, 2007). The cumulative temperature experienced during development is known to be a reliable predictor of growth and development. This metric has been widely used by agriculturists and entomologists, but has somewhat been overlooked in fisheries research (Neuheimer & Taggart, 2007). The temperature sum (degree days, DD) required to reach a particular developmental stage and the temperature threshold at which development rate is zero was estimated by linear regression for each stage (Mooij & Van Tongeren, 1990):

(3) $$Y_{\mathrm{i\tau}}=\alpha +\beta {\mathrm{Age}}_{\mathrm{i\tau}}+{\text{γStage}}_{i}$$

where *Y*_*iτ*_ is the product of the temperature *τ* and the mean age (in days) of developmental stage *i* at experimental temperature *τ* (*Age*_*iτ*_). By setting *α* to zero (no intercept), the slope *β* gives the species‐specific temperature at which development is zero (*T*_0_), and the parameter estimate *γ* of the stages represents the stage‐specific degree day metric.

### 2.8 Variation in the occurrence of smelt larvae in Lake IJsselmeer

The influence of seasonal variation in water temperature on the timing of the occurrence of smelt larvae in Lake IJsselmeer in the period 1961–2013 was studied by a simulation of the calendar date at which the degree day threshold of the 50% hatching and the 50% mortality of stage 7 as calculated earlier (Equation 3) were exceeded. Because no complete series of daily water temperatures were available for the study area, daily temperatures were estimated using a water temperature model (Mooij *et al*., 2008; Mooij & Van Tongeren, 1990):

(4) $$T_{d}=T_{d-1}+\alpha \left(A_{d}-T_{d-1}\right)+\beta +\gamma \sin\left(\frac{2\pi *\left(\left(d-1\right)-80.8\right)}{365.25}\right)$$

This model predicts the water temperature (*T*_*d*_) at day *d* of a shallow water body from the air temperature (*A*_*d*_) and the water temperature on the previous day (*T*_*d* − 1_), taking insulation and seasonal variation into account. The model was calibrated for Lake IJsselmeer using mean daily air temperatures measured at De Bilt by the Royal Netherlands Meteorological Institute of the Ministry of Infrastructure and the Environment for the period 1961–2013 and water temperature measured in Lake IJsselmeer. The parameters of the calibrated model are *α* = 0.153; *β* = 0.120; *γ* = 0.225.

With the estimated daily water temperatures, the day number was estimated at which the DD threshold at hatching (50% hatch) and at larval mortality (50% mortality) occurred. It was assumed that smelt started spawning between day 55 and day 100, as soon as the water temperature exceeded 5°C, based on variation in the timing of annual commercial fishery specifically targeting spawning smelt between 1966 and 2006 (De Leeuw, unpublished data). Similar results of water temperatures of *ca*. 5°C (ranges mentioned cover 4–8°C) at the onset of smelt spawning have been found elsewhere in temperate waters (de Groot, 2002; Hutchinson & Mills, 1987; Quigley *et al*., 2004).

## 3 RESULTS

### 3.1 Incubation experiments

**FIGURE 3:**
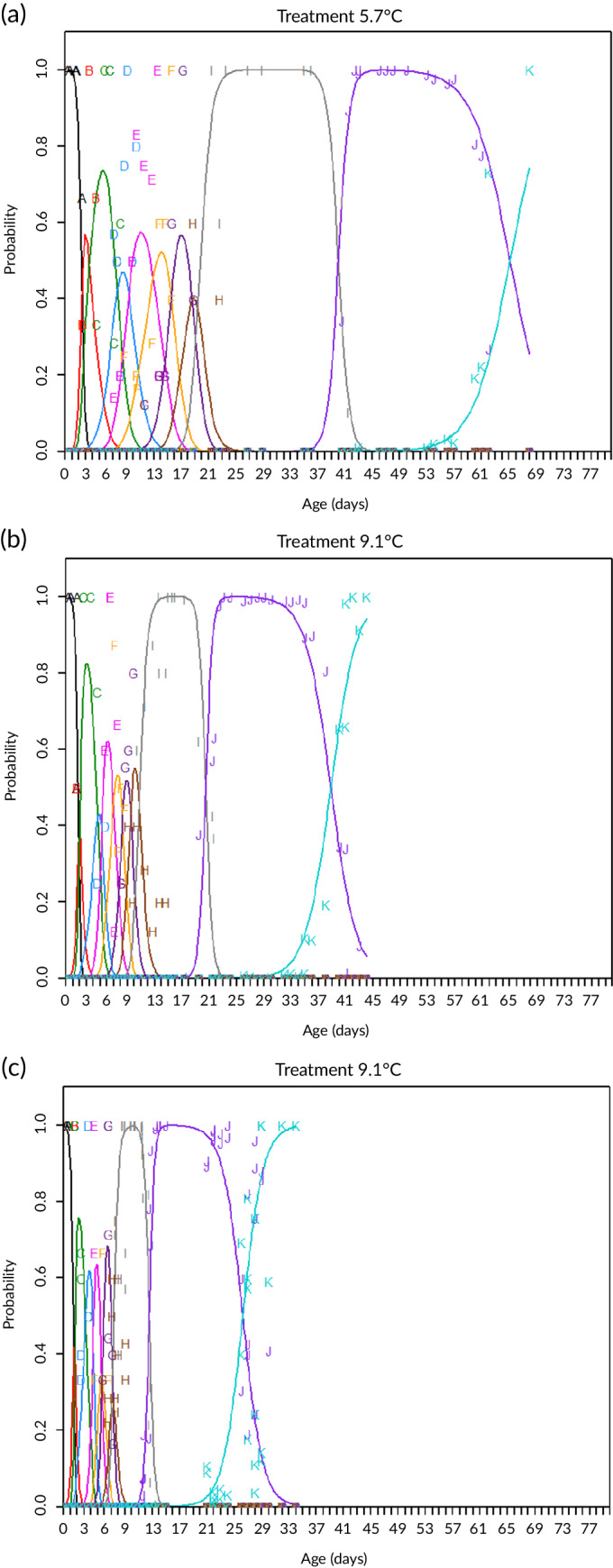
Probability, estimated by the multinomial model, of being at a particular developmental stage as a function of age at three different experimental temperatures: (a) 5.7°C; (b) 9.1°C; (c) 12.1°C. Solid lines show the predicted probability of eggs being in a certain stage at a given time under the influence of temperature. The letters refer to observations: A–I refer to stages 1–5, J refers to moment of hatching and K refers to mortality

The effect of temperature on the developmental rate differed significantly across the batches (Table 2)(Equation 1). The full model, including the main variables Age, Temperature and Batch as well as the first‐order interaction terms, had the lowest AIC and explained 79% of the variance (McFadden test, *R* ^2^ = 0.786). Batch represents the eggs from two different spawning events of the experimental population and the progeny of different parents or successive batches shed by the same parents. Because the authors were interested in the rate of development that is representative for the smelt population in Lake IJsselmeer, they used the parameter estimates of model 4 (Table 2), which ignores the effect of Batch. This model explained 78% of the variation in the observed developmental rate of smelt (McFadden test, *R* ^2^ = 0.779). The predicted probability of 50% hatching (p50‐Hatching) and 50% mortality (p50‐Mortality) after hatching with the model (4) was estimated. Due to the rapid succession of stages 1B to 4B, probabilities of eggs being at these stages were rarely above 0.8. The predicted probability of an egg being in a certain stage as a function of day number according to the model is shown in Figure 3, plotted together with the observed probability (indicated by the letters).

**FIGURE 4:**
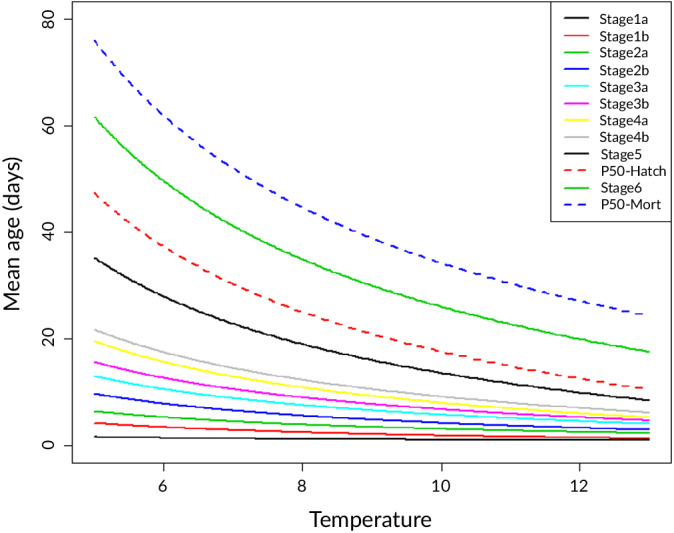
Mean age of the various egg and larval stages (solid lines) as well as the age at which 50% of the eggs hatch or 50% of the larvae died (hatched lines) estimated from Equation 2

The *T*_A_ was estimated at 11229°K (s.e. = 395) using Equation 2 and the experimental results. Figure 4 shows the model results for temperature dependence of the mean age of each developmental stage; the mean age at which 50% of the eggs hatch and at 50% mortality after hatching was calculated from Equation 2. The relationship between stage‐specific ages and temperature was curvilinear. The parameter values of the relationship for each stage are presented in Table 3. Age at 50% hatch decreased from almost 40 days at 5.7°C to 12.5 days at 12.1°C. Age at 50% mortality decreased from 66 days at 5.7°C to 26 days at 12.1°C. As a result, the endogenous feeding period of hatched larvae decreased from 25.1 days at 5.7°C to 13.7 days at 12.1°C (Figure 4).

**TABLE 3:** Estimated intercepts (*δ*) resulting from the regression of the developmental rate for each stage (Equation 2), and estimated p50‐Hatching and p50‐Mortality against temperature using Equation 3

Developmental stage	Estimated intercept *δ*	s.e.	*t*‐value	*P*
Stage 1a	39.761	1.406	28.29	<0.001
Stage 1b	39.015	1.406	27.75	<0.001
Stage 2a	38.548	1.406	27.42	<0.001
Stage 2b	38.205	1.406	27.18	<0.001
Stage 3a	37.899	1.406	26.96	<0.001
Stage 3b	37.731	1.406	26.84	<0.001
Stage 4a	37.548	1.406	26.71	<0.001
Stage 4b	37.423	1.406	26.62	<0.001
Stage 5	37.002	1.406	26.32	<0.001
Stage 6	36.382	1.406	25.88	<0.001
p50‐Hatching	36.735	1.406	26.13	<0.001
p50‐Mortality	36.130	1.406	25.7	<0.001
	Estimated Arrhenius temperature			
*T*_A_	−11,228.82	395.373	−28.4	<0.001

*Note*: Equation 2 also gives the estimate for the Arrhenius temperature *T*_A._

The temperature sums (degree days, DD) required to reach a particular developmental stage, 50% hatching and 50% mortality, were calculated with Equation 4 and tabulated in Table 4. The temperature threshold at which the development rate is zero was estimated at *T*_0_ = 1.8°C (s.e. = 0.108). The DD until 50% hatching was estimated at DD_hatch_ = 149.0 (s.e. = 3.8) ^o^Cday, and the DD till 50% mortality at DD_mort_ = 272.4 (s.e. = 5.6) ^o^Cday.

**TABLE 4:** Estimate and standard error of the degree days (DD in ^o^C days) required to reach the mean age of the developmental stages (egg stages 1–5; larval stage) and the point in time that 50% of the eggs hatch (p50‐Hatching) or 50% of the larvae died (p50‐Mortality)

Developmental stage	Estimate degree Day	s.e.	*t*‐value	*P*
Stage 1a	7.3745	2.6268	2.807	0.00725
Stage 1b	15.2184	2.6383	5.768	<0.001
Stage 2a	24.3114	2.6598	9.14	<0.001
Stage 2b	34.1926	2.6968	12.679	<0.001
Stage 3a	46.4318	2.7563	16.846	<0.001
Stage 3b	54.9311	2.8092	19.554	<0.001
Stage 4a	65.9421	2.892	22.802	<0.001
Stage 4b	74.668	2.9606	25.221	<0.001
Stage 5	113.9709	3.3776	33.743	<0.001
Stage 6	211.5162	4.6931	45.07	<0.001
p50‐Hatching	149.0169	3.837	38.837	<0.001
p50‐Mortality	272.3867	5.6094	48.559	<0.001
	Estimated temperature for which development is zero			
*T*_0_	1.7563	0.1077	16.31	<0.001

*Note: T*_0_ (°C) gives the temperature at which development stops (Equation 4).

**FIGURE 5:**
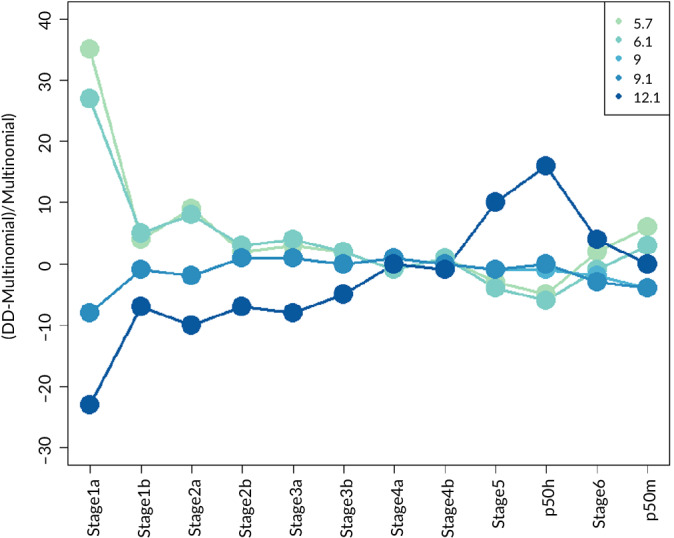
Difference (%) in the age of the various developmental stages as estimated by the degree day (DD) approach and the estimates of the multinomial analysis of the experimental observations for the five different experimental temperatures (5.7, 6.1, 9.0, 9.1 and 12.1°C) (Equation 1)

The difference between the Degree Day model and the multinomial model was least at incubation temperatures of around 9°C, except for stage 1 eggs (Figure 5). The differences between the two models increased at both higher and lower temperatures. At high temperatures, the DD overestimated age at later stages and underestimated age at early stages. At low temperatures, DD overestimated age in early stages and underestimated age at later stages.

### 3.2 Variation in development of larvae calculated from Lake IJsselmeer water temperatures

**FIGURE 6:**
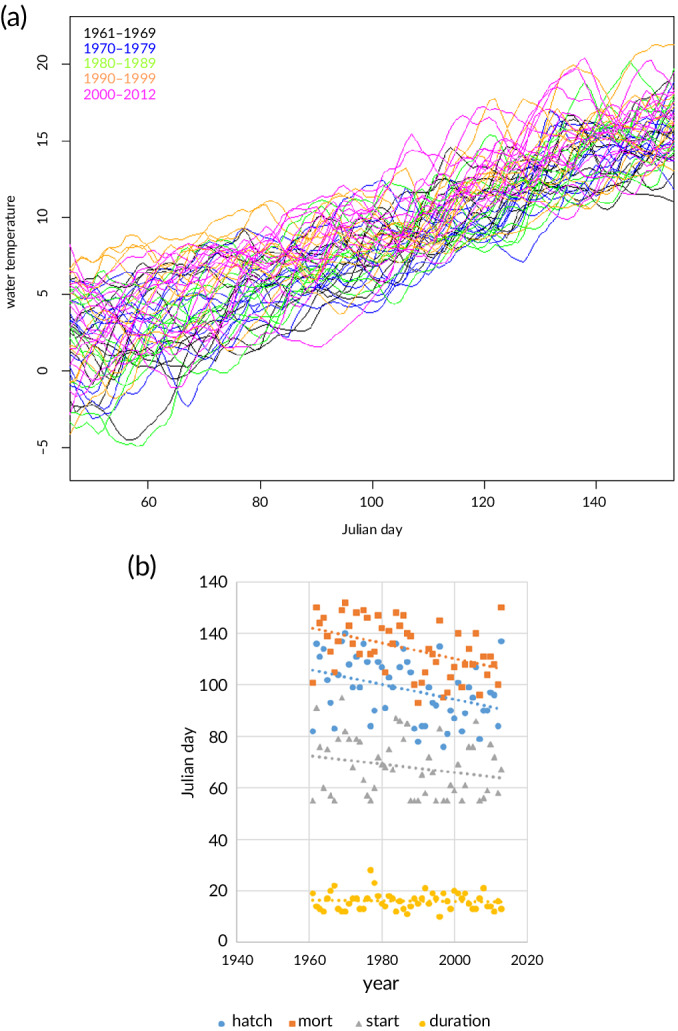
(a) Estimated water temperatures for Lake IJsselmeer from the temperature model (Equation 4) using Lake IJsselmeer data from 1961 to 2013 (different colours indicate different decades). (b) Estimated spawning day, 50% hatching, day of 50% mortality during stage 7 and the duration of the endogenous feeding period estimated from the degree days and the temperature model using Lake IJsselmeer data from 1961 to 2013. Linear trend lines were added for clarity, and the regression parameters are provided in Table 5

The estimated water temperatures for Lake IJsselmeer for a period spanning five decades are plotted in Figure 6a. For each year, the corresponding day at which the water temperature exceeded the 5°C threshold for spawning and the subsequent days when the DD thresholds for 50% hatching and 50% mortality were exceeded are plotted based on those Lake IJsselmeer temperatures (Figure 6b). In warm winters, when water temperature reached 5°C before mid‐February (Figure 6a), the start of spawning was set at day 55 irrespective of temperature, because in Lake IJsselmeer it is known that smelt does not start spawning before that date (De Leeuw, unpublished). The mean date at which smelt started spawning was estimated at day 68 (s.d. = 11.4 days) and varied by more than a month between day 55 and day 95 over the period analysed. The mean 50% hatching date of the larvae was calculated at day 98 (s.d. = 12.4 days) and varied between day 76 and day 120. The mean day of 50% larval mortality was estimated at day 114 (s.d. = 10.7 days). The endogenous feeding period duration was estimated at 16 days (s.d. = 3.5) and varied between 10 and 28 days.

The linear relationship between the onset of spawning and the 50% hatching, 50% mortality and the duration of the endogenous feeding period was established for the period 1961–2003 (Table 5). In years with relatively cold winter conditions spring temperatures showed a rapid increase compared with years with average winter conditions. Cold winter conditions led to a late onset of spawning, yet the rapid temperature increase in spring resulted in the rapid development of eggs and larvae. This was reflected in the regression slopes of the day of 50% hatching and 50% mortality as a function of the first spawning day (Table 5), which were smaller than one. Moreover, the regression slope of 50% mortality was lower than that of 50% hatching, indicating that the 50% mortality increases less rapidly with spawning day than 50% hatching. As a result, there was a negative relationship between the start of spawning and the duration of the endogenous feeding period of the larvae; in other words, late spawning is related to a shorter endogenous feeding period.

**TABLE 5:** Regression parameters of the linear relationship between the day of 50% hatching, day of 50% larval mortality and duration of the endogenous feeding period (day of 50% hatching minus day of 50% larval mortality) and the start day of spawning based on the water temperatures for 1961–2013 from Lake IJsselmeer

Dependent variable	Slope	s.e.	*P*
Hatching day	0.8035	0.102	<0.001
Mortality day	0.6288	0.097	<0.001
Duration endogenous feeding period	−0.1748	0.034	<0.001

When considering the time series, the authors found that larval presence in Lake IJsselmeer, as calculated using the temperature model, has advanced in time in response to the trend of increasing spring temperatures. The estimated day of 50% hatching and 50% mortality advanced significantly at a rate of 2.9 (s.e. = 1.05) and 3.0 (s.e. = 0.88) days per decade, respectively. The start day of spawning advanced at a rate of 1.6 (s.e. = 1.01) days per decade although this change was not significant (*P* > 0.1). The duration of the endogenous feeding period did not change significantly over time (slope = −0.1 day per decade, *P* > 0.7).

## 4 DISCUSSION

The aim of this study was to determine the effect of temperature on the development rate of smelt eggs and larvae and the duration of the endogenous feeding period. The experiments showed a significant effect of temperature on the developmental rate of stages and especially from the stage prior to hatching and onwards. By quantifying the effect of water temperature on development rate of smelt eggs and (early) larvae, the study predicted the timing and duration of the endogenous feeding period of newly hatched smelt larvae to their start of exogenous feeding based on the evolution of the temperature conditions in winter and spring since 1961.

### 4.1 Development rate of eggs

Statistical analysis showed that the developmental rate was significantly different between the batches. This result was expected because it is well established that egg size of successive batches decreases and is affected by parental effects (Chambers & Leggett, 1996; Kjesbu, 1989), and that developmental rate is affected by egg size (Pepin, 1991). As batches represented eggs produced in March or April, this could have been by different parents, or by the same parents in a second spawning event. Thus one may expect a difference in developmental rates across batches (Geffen & Nash, 2012). In this study parentage could not be identified as individuals were kept in groups (*i.e*., aquaria). Identifying differences in developmental rates between parents was not the aim of this study; in contrast, the variability was included to represent natural variability in Lake IJsselmeer. The developmental rate estimated in model 4, therefore, gives the best estimate of the effect of temperature on developmental rate in Lake IJsselmeer smelt as it ignores these parental (batch) effects. The established estimates of developmental rates presented here can be used in field research, *e.g*., to estimate the spawning time of eggs sampled in the field, or to estimate egg mortality from the decline in the density of eggs in successive sampling (Dickey‐Collas *et al*., 2003; Fox *et al*., 2000; Rijnsdorp & Jaworski, 1990).

### 4.2 Fit of the day degree model

The comparison of the predictions of the multinomial model and the degree day approach revealed differences of less than 20% in the predicted age of the hatching and larval stages, with the exception of stage 1a eggs, in particular at the lowest and highest temperatures used in the incubation experiments (5.7 and 12.1°C). The difference between the models was mainly due to the stronger curvature estimated by the degree day model (result not shown). This implies that the duration of the endogenous feeding window was overestimated by the degree day approach at low temperatures and underestimated at higher temperatures. The temperatures experienced in Lake IJsselmeer range between about 5 and 12°C for smelt eggs and between 8 and 12°C for the larvae (Figure 6), just within the temperature range over which the difference between the multinomial and degree day model converges.

### 4.3 Effect of temperature on timing of endogenous feeding

Smelt start spawning when the water temperature increases in early spring, especially at water temperatures between 4 and 6°C (Hutchinson & Mills, 1987; Power & Attrill, 2007 and references therein). Assuming that spawning commences after mid‐February at water temperatures exceeding 5°C, the predicted larval endogenous feeding period has advanced at a rate of about 2.9 days per decade since 1961. The results show that the variation in spring temperature mainly affects the timing of the endogenous feeding period, but does not affect the duration. This study's result is rather insensitive for the chosen temperature threshold for spawning. Explorations of a temperature threshold of 4 and 6°C predicted an advance of the endogenous feeding period of 2.6 and 3.3 days per decade, respectively.

### 4.4 Implications for first feeding

The importance of the timing of the endogenous feeding period and therefore first feeding of larvae to match with the peak in their planktonic food is central to the match–mismatch hypothesis (Cushing, 1990) and has been supported in several species (Durant *et al*., 2007; Kristiansen *et al*., 2011; Platt *et al*., 2003). For rainbow smelt, *O. mordax*, it was shown that strong year classes developed when there was overlap both temporally and spatially with abundant zooplankton (O'Brien *et al*., 2012). Nonetheless, it is the degree in overlap between larvae and their food, and not necessarily an overlap with peak abundances, that matters for survival (Kristiansen *et al*., 2011). Still, given that day length will trigger the end of zooplankton hibernation phase [but temperature determines the subsequent growth and development of the zooplankton stages (de Senerpont Domis *et al*., 2007)], a mismatch between the occurrence of smelt larvae and their zooplankton prey may occur with early hatching smelt, given the difference in environmental cue to which smelt and zooplankton appear to respond. Zooplankton in Lake IJsselmeer first appears in April (Noordhuis, 2010). Given the time frame of the critical start of the exogenous feeding period and the more frequent occurrence of 50% hatching before 1 April (Julian day 90) (Figure 6) in later decades, mismatches between smelt larvae and their zooplankton prey are possible in years with advanced recruitment and further increase in water temperatures. A smelt larvae‐zooplankton mismatch could therefore contribute to the gradual decrease in smelt stocks observed since 1990 in Lake IJsselmeer and Lake Markermeer. Similarly, lower recruitment of smelt stocks has been observed in the River Thames, UK (Power & Attrill, 2007), in France (Pronier & Rochard, 1998) and in Lake Peipsi, Estonia/Russia (Kangur & Kangur, 2007) in recent decades, presumably related to higher water temperatures.

In contrast to negative effects on recruitment of smelt when mismatches occur with prey availability, higher spring temperatures might also have positive effects on larval survival. Higher growth rates reduce development time of eggs and larvae, thereby reducing predation pressure on the larvae. Simonin *et al*. (2016; Simonin et al. 2019), for example, argued that rainbow smelt in the great lakes of North America is cannibalistic and incurs major predation risks for larvae, but that temperature‐induced higher growth rates of larvae might lead to higher recruitment because of reduced predation pressure. Similarly, smelt in Lake IJsselmeer experiences considerable predation by young‐of‐the‐year perch (*Perca fluviatilis*) and pikeperch (*Sander lucioperca*). Especially late‐hatching smelt are preyed upon by early hatching perch and pikeperch, whereas late‐hatching pikeperch are not able to switch to piscivory (Buijse *et al*., 1993).

Relationships between water temperature changes and recruitment of smelt are therefore not straightforward: timing of hatching and development time vary seasonally and between years and interact with both seasonal dynamics of smelt prey (zooplankton) and smelt predators (larger smelt and other piscivores). Indeed, Henderson and Nepszy (1989) found that recruitment of young‐of‐the‐year rainbow smelt was on average not dependent on water temperature (rate of warming and average temperature) during spawning. Arula *et al*. (2017), on the contrary, demonstrated that changed climatic conditions increased the variability in annual recruitment and, in turn, made smelt populations more vulnerable to external conditions. In addition to effects on smelt larvae, higher temperatures in summer seem to affect smelt populations negatively (Arula *et al*., 2017; Kangur & Kangur, 2007; Power & Attrill, 2007; Simonin *et al*., 2016).

### 4.5 Implications for smelt fishery

Sensitivity of smelt to higher temperatures, at both larval and juvenile stages, ultimately results in unstable smelt populations and uncertainties for smelt fishery with implications for its performance (Arula *et al*., 2017; Kangur & Kangur, 2007). Unstable smelt stocks were observed in Lake IJsselmeer the last decade, strongly reducing the options for a smelt fishery because of uncertain catches and therefore an uncertain market. In addition, unstable smelt stocks led to fewer fish‐eating birds that are protected under the European Bird and Habitat Directive, further delimiting options for smelt fishery. Indeed, smelt fishery in Lake IJsselmeer has been closed in most years since 2003.

### 4.6 Conclusion

Larvae are present earlier in the season with increasing water temperature, but the duration of the endogenous feeding period has not changed during the past 50 years. Given global warming of waters and the temporal advancement of the critical period between hatching and yolk‐sac depletion, timing mismatches between fish larvae and their zooplankton prey may occur more frequently and could result in recruitment failure.

## AUTHOR CONTRIBUTIONS

M.K. developed the methodology, performed the formal analysis and project administration and drafted the original manuscript. P.M. performed the experiments and commented on the manuscript. Jd.L. acquired funding, developed the methodology and edited the manuscript. W.M. acquired funding and supervised the project. A.R. acquired funding, supervised the project and drafted the original manuscript. Kvd.W. performed the formal analysis, wrote and edited the final manuscript.

## Supporting information

Supporting Information Table S1 Description of each stage used in the smelt experiments and modelling.
