# Supplementary material for: Temperature effects on egg and larval development rate in European smelt, Osmerus eperlanus, experiments and a 50 year hindcast
Source: J Fish Biol. 2020 Apr 5;96(6):1422–33. doi: 10.1111/jfb.14314 (PMC7383854; doi:10.1111/jfb.14314)
Supplement: Supplementary file 1 — Supporting Information Table S1 Description of each stage used in the smelt experiments and modelling. [file JFB-96-1422-s001.docx]

Supporting Information - Egg development stages

Table S1. Description of each stage used in the smelt experiments and modelling.

| Stage | Description |
| --- | --- |
| 1a | Stage 1a starts directly after fertilization, when the zygotic nucleus is formed. This stage includes the 10-11 cycles of cell divisions that lead to the formation of the blastula. |
| 1b | Stage 1b represents gastrulation up until the epibolia and embryonic band is formed. |
| 2a | Stage 2a begins with the start of somitogenesis when the first pairs of somites are formed. |
| 2b | Stage 2b, continues into somitogenesis. The embryo is now surrounding half of the yolk sac. Furthermore, the optic cups, Kupfer’s vesicle, segments of the hindbrain and the notochord are formed. |
| 3a | Stage 3a, still during somitogenesis: the embryo surrounds the yolk completely. The lenses as well as the otic vesicle (auditory vesicle), the non-vacuolated notochord and the intestinal canal are also formed. |
| 3b | Stage 3b: the first pigmentation of the eyecups becomes visible. Development of the gill region and, most notably, the first signs of a pulsating heart are also included in this stage. |
| 4a | Stage 4a: the formation of an early stomach, dense pigmentation of the optic cups become visible and the hatching glands on the dorsal side of the head appear. |
| 4b | Stage 4b, the final stage of somitogenesis: the cavity for the intestinal canal develops and the origin of the liver separates and differentiates. In addition, there are multiple melanophores present on the yolk sac and along the fold of the ventral fin, and the pectoral fins start to form. |
| 5 | Stage 5, final stage before hatching: a conglomerate chain of melanophores along the intestines and caudal part is visible. Furthermore, the liver is now situated at the 9^th^-18^th^ segment. During this stage, the anal canal and the ventral mouth position are completed. |
| 6 | Stage 6 is hatching (henceforth referred to as hatch) during which the larvae break through the zona radiata interna with intense posterior movements that result in the detachment of the larvae from the egg. |
| 7 | Stage 7 refers to the larval yolk sac stage of the now free swimming larvae. |
| 50% hatch | The index of 50% hatching refers to the probability of half of the eggs reaching stage 6 and becoming free swimming larvae. |
| 50% mort | The index of 50% mortality refers to the probability that half of the larvae in stage 7 have died. |
